# Fungicidal Properties of the Essential Oil of *Hesperozygis marifolia* on *Aspergillus flavus* Link

**DOI:** 10.3390/molecules16032501

**Published:** 2011-03-15

**Authors:** Marco Martín González-Chávez, Norma Cecilia Cárdenas-Ortega, Cristina Andrea Méndez-Ramos, Salud Pérez-Gutiérrez

**Affiliations:** 1 Centro de Investigación y Estudios de Posgrado, Facultad de Ciencias Químicas, Universidad Autónoma de San Luis Potosí, Av. Dr. Manuel Nava 6, Zona Universitaria, San Luis Potosí, SLP, 78210, Mexico; 2 Departamento de Sistemas Biológicos, Universidad Autónoma Metropolitana, A.P. 23-181, México D. F. 11560, Mexico

**Keywords:** essential oil, *Hesperozygis marifolia*, (*R*)-pulegone, fungicidal activity, *Aspergillus flavus*

## Abstract

The chemical composition of the essential oil from *Hesperozygis marifolia* was analyzed by gas chromatography-mass spectrometry (GC-MS), and fourteen compounds were identified. (*R*)-pulegone (40.75%), isomenthone (30.34%) and menthone (4.46%) were found to be the main components of the oil. The essential oil at a concentration of 2.0 mg/mL and (*R*)-pulegone at concentration of 0.8 mg/mL completely inhibited the growth of *Aspergillus flavus* Link. The fungicidal effects of this essential oil warrant further research into its potential for commercial use.

## 1. Introduction

Grain silage preservation relies on anaerobic conditions during storage. Poor storage conditions (insufficient drying, condensation, heating, rainwater leakage, or insect infestation) can lead to the growth of anaerobic and microaerobic acid-tolerant fungi. Among these fungi, the toxigenic, *Aspergillus flavus* Link produces carcinogenic aflatoxins, that affect the liver and lungs tissues [1,2]. Synthetic fungicides are used to control the pathogenic fungi, although the fungi can produce resistant plaques and that are carcinogenic and teratogenic [3]. 

There is a clear need for new methods of preserving grains using natural additives. One approach involves introducing essential oils as antifungal additives [4,5,6,7]. These oils are rich sources of biologically active compounds. *Hesperozygis marifolia* Epling (Lamiaceae) is a shrub, 20–50 cm long, with leaves up to 15 mm in length. It is used in traditional medicine to treat gastrointestinal problems and as a tranquillizer [8]. The compounds 2-hydroxy-3-(1’,1’-dimethylalyl)-1,4-naphthoquinone, α-dunione, betulin acetate and 5-*O*-desmethylnobiletine have been isolated from the roots of *H. marifolia* by acetone extraction [9].

The aims of this study were to investigate the chemical composition of the hydrodistilled essential oil from *H. marifolia* using gas chromatography-mass spectroscopy (GC-MS) analysis and to determine the *in vitro* antifungal activity of the essential oil and its major components against *A. flavus*.

## 2. Results and Discussion

Hydrodistillation of the aerial parts of *H. marifolia* produced a colorless oil in 2.0% yield. Optical analysis yielded η_D_^25° ^= 1.4580 and ρ^25 °C ^= 0.8951 g/mL. GC/MS analysis of the crude oil identified 14 compounds (Table 1) representing 84.69% of the oil. (*R*)-Pulegone (40.75%) and isomenthone (30.34%) were the main components, followed by menthone (4.46%). The composition of the oil was determined by comparing the relative retention times to the retention times of standardized samples and the mass spectra of the components to the mass spectra provided in the data library.

**Table 1:** Composition of the essential oil isolated from *H. marifolia*.

Compound	Composition* (%)	Retention time (min)
α-Pinene	0.16	3.92
β-Pinene	0.12	5.74
Limonene	0.41	8.52
Cyclohexanone, 3-methyl-	0.16	13.17
Tyranton	3.44	14.97
3–Octanol	0.38	16.72
Menthone	4.46	18.68
1–Octen-3–ol	1.06	18.90
Isomenthone	30.34	19.82
Isopulegone	0.49	22.90
Caryophyllene	1.38	23.68
(*R*)-Pulegone	40.75	25.73
Borneol	1.43	28.14
*cis*-Jasmone	0.11	36.03

*Values represent the relative abundance (%) of each compound

The absolute structure of the pulegone was established by HPLC analysis on an OJ-H chiral column. The retention time of the most abundant peak (the essential oil) was 6.8 min, which agreed with the retention time of (*R*)-pulegone. A retention time of 7.2 min was measured for (*S*)-pulegone. The intensity of the peak at 6.8 min increased upon addition of the standard (*R*)-pulegone sample, indicating that the essential oil was enriched with (*R*)-pulegone. This result confirmeds that the *H. marifolia* essential oil contained only the (*R*)-pulegone, as it has been found in the species *H. ringens* and *H. rhododon* Epling [10]. 

In this study the essential oil of *H. marifolia* was tested for its fungicidal properties toward the fungus *A. flavus*. The oil was both fungistatic and fungicidal against *A. flavus* at concentrations of 1.0 and 2.0 mg/mL, respectively, in liquid culture media. 

The effects of some essential oils on the growth of *A. flavus* have previously been demonstrated [11]. The essential oil isolated from *Chyisactinia mexicana* has been found to be fungistatic and fungicidal against *A. flavus* at concentrations of 1.0 and 1.25 mg/mL, respectively.

These results indicate that the efficacy of the essential oil depends on its chemical composition. To verify this assertion, pure commercial (*R*)-pulegone, menthone, and isomenthone, the major constituents of the *H. marifolia* essential oil, were tested individually against *A. flavus*, under identical conditions. The activities of these components were compared with the activity of the essential oil. Menthone and isomenthone did not display any activity against *A. flavus*, while (*R*)-pulegone strongly inhibited mycelial growth. Complete growth inhibition was observed at concentration (0.8 mg/mL), lower than the essential oil MIC. 

Oumzil *et al.* [12] found that pulegone showed antimicrobial activity toward several bacteria and three fungi. Similar results were obtained by others [13,14] who found that pulegone showed more potent biocide activity than menthone. At a concentration of 1.0 µL/45 mL, pulegone resulted in 100% mortality in *Frankliniella occidentalis*. This compound also showed insecticidal activity toward *Moechotypa* diphysis [15]. 

This study showed that the essential oil and (*R*)-pulegone may be suitable for use as fungicide. Further research is required, including an investigation of the oil’s sorption, adsorption, and residual properties on a target commodity and the influence the residues on the product’s acceptability, worker safety and mammalian consumption.

## 3. Materials and Methods

### 3.1. Collection of the plant material

The aerial parts of *H. marifolia* were collected during flowering time from the hills surrounding the region of Las Comadres, Municipality of Guadalcazar, San Luis Potosí State, Mexico, in August 2007. The plant material was taxonomically identified using the species classification system developed in the Isidro Palacios Herbarium of the Universidad Autónoma de San Luis Potosí. A voucher specimen (SPLM43012) was deposited in the Herbarium archive. 

### 3.2. Isolation of the essential oil

The fresh aerial parts (leaves and flowers) of the plant were submitted to hydrodistillation in a Clevenger-type apparatus. The aqueous phase was saturated with sodium chloride and extracted with diethyl ether. The ether was dried over anhydrous sodium sulfate and concentrated at room temperature. The oil was stored at 4 °C until testing and analysis. The refractive index of the oil was determined at 25 °C using a Zeiss Opton refractometer.

### 3.3. GC-MS analysis

The essential oil was analyzed using an Agilent Technology GC 6890N coupled with a MS 5973N mass spectrometer operated at 70 eV and equipped with a Carbowax capillary column (30 m, 0.25 i.d.) with a polyethylene glycol matrix (0.25 µm thick). Helium was used as the carrier gas at a flow rate of 1 mL/min. The injector and MS transfer line temperatures were set at 250 °C. Diluted samples (1:10 v/v, in acetone) 1.0 µL in volume were manually injected. After sample injection, the initial temperature in the oven (45 °C) was held constant for 3 min, then increased to 135 °C (at a rate of 3 °C/min). This temperature was maintained for 1 min, then increased to 250 °C (at a rate of 10 °C/min). This temperature was then maintained for 10 min. After a delay of 3 min to permit passage of the solvent, the mass spectra were scanned from 15 to 800 *m/z*. The compounds were identified by comparing the relative retention times and mass spectra with those of commercial compounds, with reference to the mass spectra reported in the U.S. National Institute of Standards and Technology Library NIST02.L.

### 3.4. HPLC analysis

The chromatography instrument used for this purpose was an Agilent 1100 equipped with a UV-vis photodiode array detector. The essential oil and pulegone (obtained from Sigma-Aldrich) were analyzed by analytical HPLC using an OJ-H chiral column (Chirasel 4.6 × 260 mm) at 25 °C. The elution profile was measured under constant flow provided by a solution containing 95:5 hexane-isopropanol. The flow rate of the mobile phase was 0.6 mL/min, the injection volumes were 5 μL, and the eluent was analyzed by absorption at λ = 230 nm.

### 3.5. Minimum inhibitory concentration (MIC)

The MIC was determined by the serial dilution method. The essential oil was diluted with DMSO and added to 4 mL of a Sabouraud dextrose broth to obtain a final concentration between 0.5 and 2.0 mg/mL. These solutions were placed in culture tubes and the tubes were inoculated with *A. flavus* Link spores (SRRC 1273 from the National Center for Agricultural Utilization, Peoria IL, USA, 1,000 spores per 0.1 mL). Tubes were incubated at 28 ± 1 °C for 72 h. The MIC was determined by the concentration at which no fungus growth was observed. 

### 3.6. Fungicidal or fungistatic activity

Tubes of Sabouraud dextrose broth containing the essential oil serial dilutions (0.2 to 2.0 mg/mL) were inoculated with 0.1 mL spore suspension and incubated for 72 h at 28 ± 1 °C. Following incubation a 0.1 mL spore suspension sample was dispersed onto each plate containing Czapek agar, followed by incubation for 72 h at 28 ± 1 °C. The concentration at which the growth of *A. flavus* was inhibited in the Sabouraud dextrose broth but not on the Czapek agar determined the fungistatic concentration. Then concentration at which neither media supported growth determined the minimum fungicidal concentration (MFC). The MIC and MFC were determined for the essential oil and its main components [(*R*)-pulegone, menthone, and isomenthone] in DMSO. Each isolate was diluted in 40 mL broth to a final concentration in the range 0.4–1.0 mg/mL.

### 3.7. Statistical analysis

Experimental values of the MIC and MFC are expressed as mean of at least three experiments in duplicate. Data were analyzed by using one ANOVA. The level of ρ 0.05 was used as criterion of statistical significance. All calculations were done using STATISTICA V 7.1 (StatSoft Inc., Tulsa, OK, USA).

## 4. Conclusions

This study constitutes the firt report of the fungicidal activity of (*R*)-pulegone against *A. flavus*. The efficacy of the essential oil of *H. marifolia* and its main component (*R*)-pulegone against this fungus may contribute to the protection of grains during storage.
